# Supplementary material for: Crop calendar optimization for climate change adaptation in yam farming in South-Kivu, eastern D.R. Congo
Source: PLoS One. 2024 Sep 4;19(9):e0309775. doi: 10.1371/journal.pone.0309775 (PMC11373801; doi:10.1371/journal.pone.0309775)
Supplement: S1 Fig — (DOCX) [file pone.0309775.s001.docx]

**
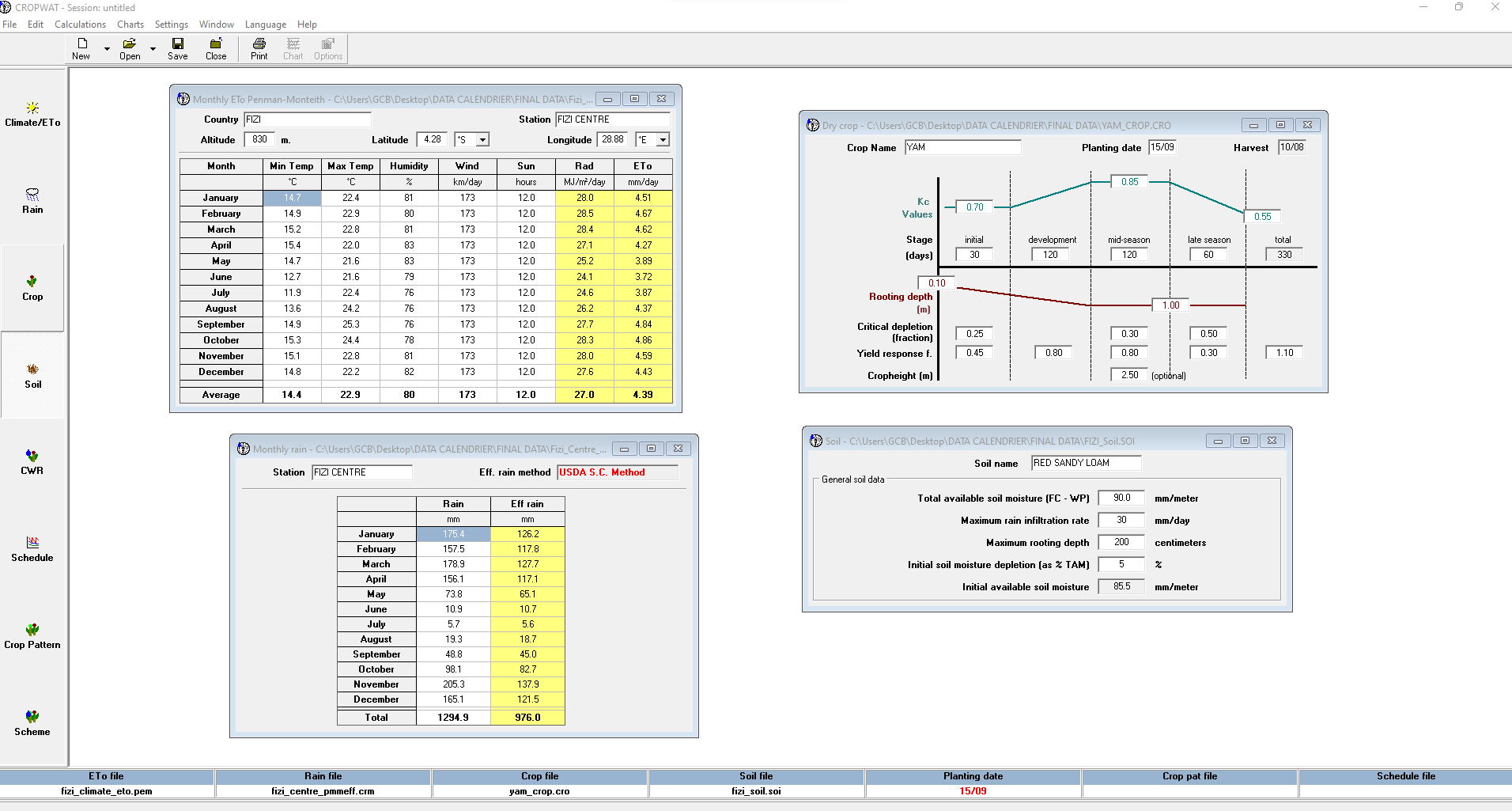
**

**S1 Fig. Different windows of CROPWAT 8.0 used for parametrization and assessment of the water demand of yam in the different agroecological zones in South-Kivu.** Simulated values are presented in yellow column with the input values
