# Supplementary material for: Crop calendar optimization for climate change adaptation in yam farming in South-Kivu, eastern D.R. Congo
Source: PLoS One. 2024 Sep 4;19(9):e0309775. doi: 10.1371/journal.pone.0309775 (PMC11373801; doi:10.1371/journal.pone.0309775)
Supplement: S2 Fig — (DOCX) [file pone.0309775.s002.docx]

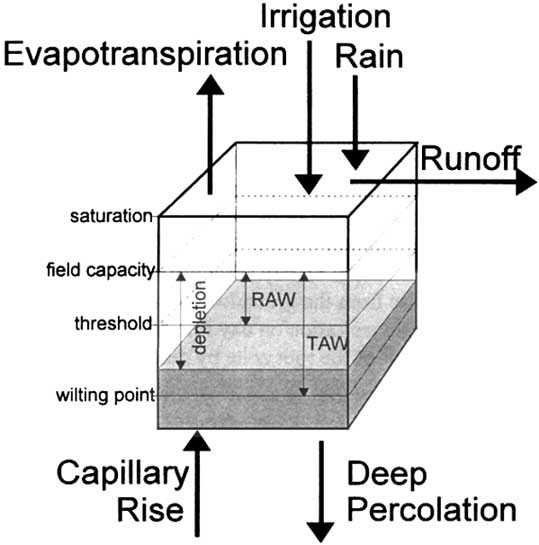


**S2 Fig. Diagram of the water balance elements used in the soil parameterization of the CROPWAT tool**. The darker area represents a certain level of water retained by the soil (adapted from Surendran et al., 2017)
