# Supplementary material for: Crop calendar optimization for climate change adaptation in yam farming in South-Kivu, eastern D.R. Congo
Source: PLoS One. 2024 Sep 4;19(9):e0309775. doi: 10.1371/journal.pone.0309775 (PMC11373801; doi:10.1371/journal.pone.0309775)
Supplement: S3 Fig — (DOCX) [file pone.0309775.s003.docx]

**(a)**

**(b)**

**(c)**

**S3 Fig. Umbrothermal diagrams of South-Kivu AEZs:** (**a**) Low altitude with semi-arid climate AEZ1 (Uvira and northern Fizi), (**b**) Typical equatorial or subtropical AEZ 2 (western Kalehe, southern Fizi, Mwenga, and Shabunda)**,** and (**c**) Medium altitudes with humid tropical (AEZ3) or mountainous tropical climate AEZ4 (in Kabare, Walungu, Idjwi, and southern Kalehe)
