# Supplementary material for: Crop calendar optimization for climate change adaptation in yam farming in South-Kivu, eastern D.R. Congo
Source: PLoS One. 2024 Sep 4;19(9):e0309775. doi: 10.1371/journal.pone.0309775 (PMC11373801; doi:10.1371/journal.pone.0309775)
Supplement: S5 Fig — Calculation of SPI for equatorial (AEZ2) and humid tropical climates temperate with altitude (AEZ4) for each year (a) and each month (b). (DOCX) [file pone.0309775.s005.docx]

**(a)**

**(b)**

**S5 Fig. Calculation of SPI for equatorial (AEZ2) and humid tropical climates temperate with altitude (AEZ4) for each year (a) and each month (b).** The bar above each column represents the standard deviation of the monthly mean values for each year. Red and blue values represent the humid and dry year or month, respectively.
