# Supplementary material for: Crop calendar optimization for climate change adaptation in yam farming in South-Kivu, eastern D.R. Congo
Source: PLoS One. 2024 Sep 4;19(9):e0309775. doi: 10.1371/journal.pone.0309775 (PMC11373801; doi:10.1371/journal.pone.0309775)
Supplement: S7 Fig — Distribution of crop water demand (ETc) and effective rainfall per month (a) and decade (b) throughout the year in the AEZ 2. (DOCX) [file pone.0309775.s007.docx]

**(a)**

**(b)**

**S7 Fig. Distribution of crop water demand (ETc) and effective rainfall per month (a) and decade (b) throughout the year in the AEZ 2.** Favorable periods, in terms of water availability, are those where effective rainfall exceeds crop water demand, i.e., when the green curve is above the red one. The vertical lines correspond to periods of changing conditions. The greater the difference between the two curves, the greater the gap (translated into water shortage or flooding), and the more actions are required.
