# Supplementary material for: Crop calendar optimization for climate change adaptation in yam farming in South-Kivu, eastern D.R. Congo
Source: PLoS One. 2024 Sep 4;19(9):e0309775. doi: 10.1371/journal.pone.0309775 (PMC11373801; doi:10.1371/journal.pone.0309775)
Supplement: S10 Fig — Distribution of plant water demand (ETc) and effective rainfall by month (a) and decade (b) of the year. (DOCX) [file pone.0309775.s010.docx]

**(a)**

**(b)**

**S10 Fig. Distribution of plant water demand (ETc) and effective rainfall by month (a) and decade (b) of the year.** Favorable periods, in terms of water availability, are those where effective rainfall exceeds crop water demand, i.e., when the green curve is above the red one). The vertical lines correspond to periods of changing conditions. The greater the difference between the two curves, the greater the gap (translated in water shortage or flooding), and the more actions are required.
