# Supplementary material for: Crop calendar optimization for climate change adaptation in yam farming in South-Kivu, eastern D.R. Congo
Source: PLoS One. 2024 Sep 4;19(9):e0309775. doi: 10.1371/journal.pone.0309775 (PMC11373801; doi:10.1371/journal.pone.0309775)
Supplement: S11 Fig — (DOCX) [file pone.0309775.s011.docx]

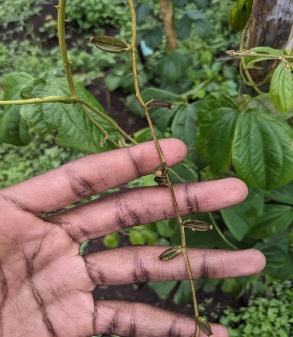

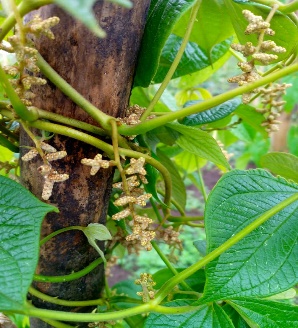

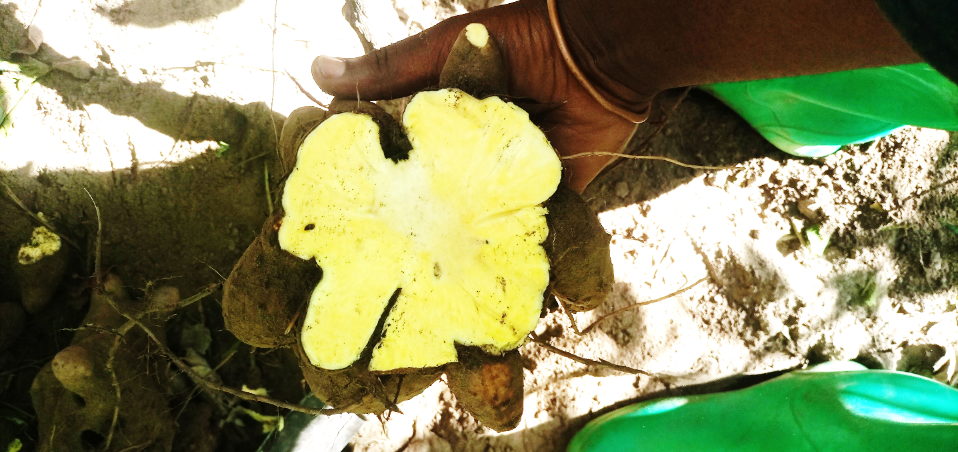

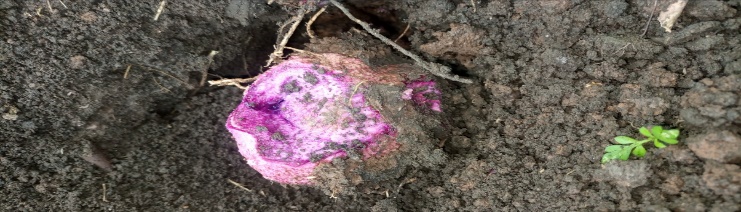

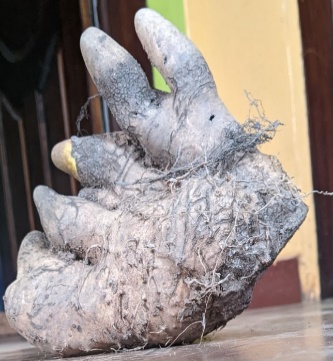

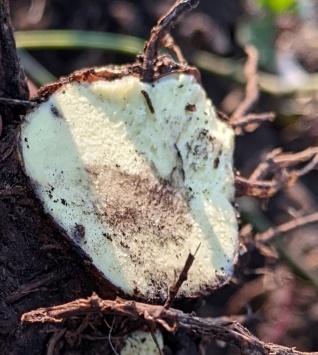

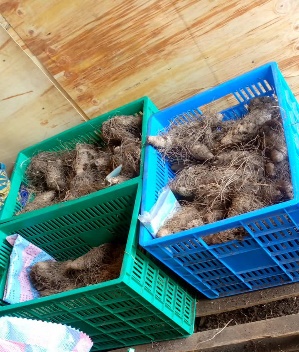

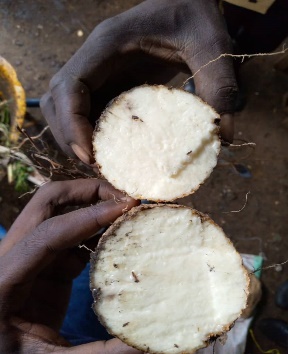

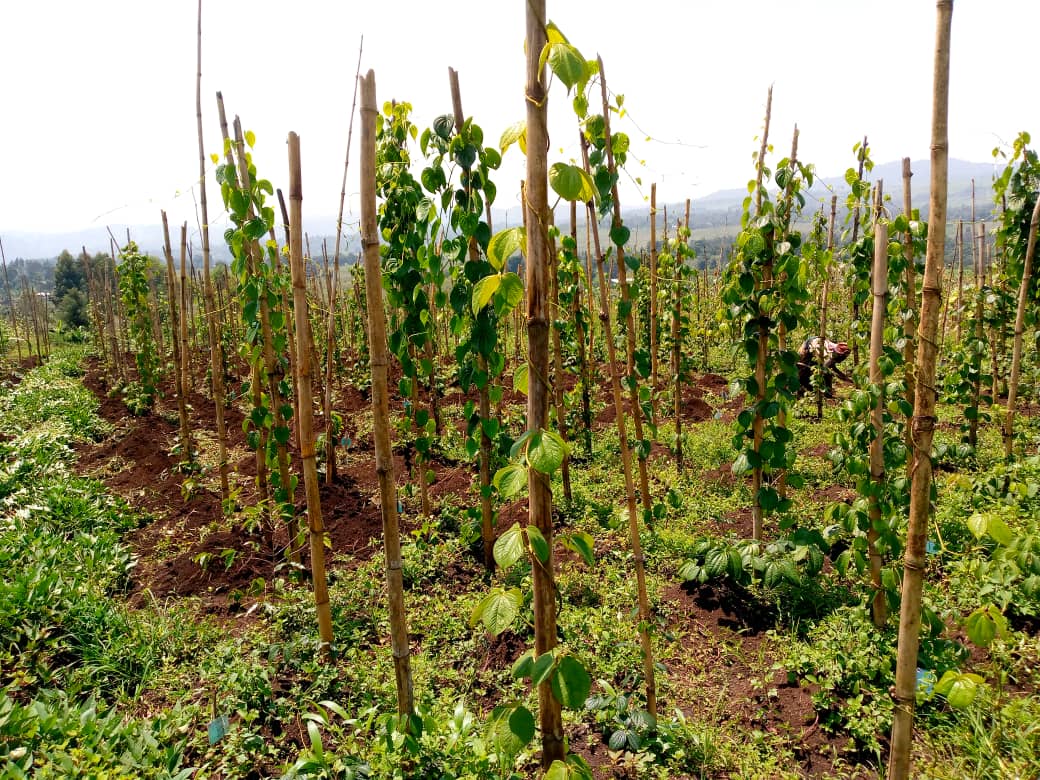


B

C

D

E

F

G

H

I

A

**S11 Fig.** **Pictures of the yam crop in the field settings in South-Kivu, eastern DRC**. (**a**), (**b**), (**f**), and (**h**) show tuber flesh color diversity; (**c**) and (**d**) illustrate flowering stage; (**e**) and (**f**) show tuber shape while (**i**) shows a yam field at vegetative stage being weeded.

A
