# Supplementary material for: Crop calendar optimization for climate change adaptation in yam farming in South-Kivu, eastern D.R. Congo
Source: PLoS One. 2024 Sep 4;19(9):e0309775. doi: 10.1371/journal.pone.0309775 (PMC11373801; doi:10.1371/journal.pone.0309775)
Supplement: S1 Table — (DOCX) [file pone.0309775.s012.docx]

**S1 Table. Indices names, definition and units used for each indicators**

| **ID** | **Indicator name** | **Definitions** | **Units** |
| --- | --- | --- | --- |
| FD0 | Frost days | Annual count when TN(daily minimum)<0ºC | Days |
| SU25 | Summer days | Annual count when TX(daily maximum)>25ºC | Days |
| ID0 | Ice days | Annual count when TX(daily maximum)<0ºC | Days |
| TR20 | Tropical nights | Annual count when TN(daily minimum)>20ºC | Days |
| GSL | Growing season Length | Annual (1st Jan to 31st Dec in NH, 1st July to 30th June in SH) count between first span of at least 6 days with TG>5ºC and first span after July 1 (January 1 in SH) of 6 days with TG<5ºC | Days |
| TXx | Max Tmax | Monthly maximum value of daily maximum temp | ºC |
| TNx | Max Tmin | Monthly maximum value of daily minimum temp | ºC |
| TXn | Min Tmax | Monthly minimum value of daily maximum temp | ºC |
| TNn | Min Tmin | Monthly minimum value of daily minimum temp | ºC |
| TN10p | Cool nights | Percentage of days when TN<10th percentile | Days |
| TX10p | Cool days | Percentage of days when TX<10th percentile | Days |
| TN90p | Warm nights | Percentage of days when TN>90th percentile | Days |
| TX90p | Warm days | Percentage of days when TX>90th percentile | Days |
| WSDI | Warm spell duration indicator | Annual count of days with at least 6 consecutive days when TX>90th percentile | Days |
| CSDI | Cold spell duration indicator | Annual count of days with at least 6 consecutive days when TN<10th percentile | Days |
| DTR | Diurnal temperature range | Monthly mean difference between TX and TN | ºC |
| RX1day | Max 1-day precipitation amount | Monthly maximum 1-day precipitation | mm |
| Rx5day | Max 5-day precipitation amount | Monthly maximum consecutive 5-day precipitation | mm |
| SDII | Simple daily intensity index | Annual total precipitation divided by the number of wet days (defined as PRCP>=1.0mm) in the year | mm/day |
| R10 | Number of heavy precipitation days | Annual count of days when PRCP>=10mm | Days |
| R20 | Number of very heavy precipitation days | Annual count of days when PRCP>=20mm | Days |
| Rnn | Number of days above in mm | Annual count of days when PRCP>=nn mm, nn is user defined threshold | Days |
| CDD | Consecutive dry days | Maximum number of consecutive days with RR<1mm | Days |
| CWD | Consecutive wet days | Maximum number of consecutive days with RR>=1mm | Days |
| R95p | Very wet days | Annual total PRCP when RR>95th percentile | mm |
| R99p | Extremely wet days | Annual total PRCP when RR>99th percentile | mm |
| PRCPTOT | Annual total wet-day precipitation | Annual total PRCP in wet days (RR>=1mm) | m |
