# Supplementary material for: Crop calendar optimization for climate change adaptation in yam farming in South-Kivu, eastern D.R. Congo
Source: PLoS One. 2024 Sep 4;19(9):e0309775. doi: 10.1371/journal.pone.0309775 (PMC11373801; doi:10.1371/journal.pone.0309775)
Supplement: S2 Table — (DOCX) [file pone.0309775.s013.docx]

**S2 Table.** **Estimated crop water requirement (ETc), effective rainfall (Eff rain), and the cumulative water deficit (in mm/dec) for yam across South-Kivu AEZs**

| **Zone** | **Stage** | **ETc (mm/dec)** | **Eff rain (mm/dec)** | **Cumulative deficit (mm/dec)** |
| --- | --- | --- | --- | --- |
| **AEZ 1** | *Initial* | 27.3 | 22.1 | 7.8 |
|  | *Development* | 34.3 | 41.1 | 0.4 |
|  | *Mid* | 33.0 | 28.5 | 7.6 |
|  | *Late* | 26.3 | 3.8 | 22.4 |
| ***Mean*** |  | **31.6** | **27.7** | **7.9** |
| **AEZ 2** | *Initial* | 26.1 | 37.4 | 0.3 |
|  | *Development* | 30.8 | 49.2 | 0.0 |
|  | *Mid* | 29.6 | 39.2 | 4.0 |
|  | *Late* | 22.6 | 20.4 | 12.6 |
| ***Mean*** |  | **28.4** | **39.1** | **3.8** |
| **AEZ 3** | *Initial* | 32.1 | 31.9 | 4.7 |
|  | *Development* | 39.0 | 40.5 | 1.6 |
|  | *Mid* | 39.0 | 27.9 | 11.6 |
|  | *Late* | 33.1 | 11.1 | 22.0 |
| ***Mean*** |  | **37.3** | **29.8** | **9.2** |
| **AEZ 4** | *Initial* | 28.79 | 31.97 | 1.05 |
|  | *Development* | 35.94 | 41.97 | 0.22 |
|  | *Mid* | 36.39 | 33.91 | 4.83 |
|  | *Late* | 28.75 | 12.51 | 16.17 |
| ***Mean*** |  | **27.7** | **7.9** | **31.6** |

*AEZ : agroecological zone, ETC : cultural evapotranspiration, Eff rain : effective rainfall, dec : decade*
