# Supplementary material for: Crop calendar optimization for climate change adaptation in yam farming in South-Kivu, eastern D.R. Congo
Source: PLoS One. 2024 Sep 4;19(9):e0309775. doi: 10.1371/journal.pone.0309775 (PMC11373801; doi:10.1371/journal.pone.0309775)
Supplement: Attachment — Submitted filename: RESPONSE TO REVIEWERS_R1.docx [file pone.0309775.s014.docx]

**RESPONSE TO REVIEWER COMMENTS**

**PONE-D-24-20765: Crop calendar optimization for climate change adaptation in yam farming in South-Kivu, eastern D.R. Congo**

**PLOS ONE**

==============================

**General comments/Academic editor**

**Comment 1.** The work presented is very important, but few minor concerns are noted below:

**Response 1.** Dear Editor, many thanks for sparing part of your valuable time to add value to our manuscript. Thank you for supporting its relevance as well. All your concerns have been addressed in the revised manuscript.

**Comment 2.** Please provide further justification of why CROPWAT was used over another model such as AquaCrop which can do the same type of analysis.

**Response 2.** Thank you for your comment. As far as the choice of model is concerned, we preferred to use CROPWAT because it is convenient and well adapted to local conditions. In fact, previous studies elucidated the effectiveness of CROPWAT to simulate the crop calendar in the study area. This research being complementary to previous efforts, we selected CROPWAT to facilitate comparison of outcomes. Based on literature, CROPWAT is the most widely used for determining crop calendar while AquaCrop is mainly used to assess the productivity of crops such as rice, maize, sorghum, etc. This information has been added to the revised manuscript.

**Comment 3.** You also mentioned that you have used NASA power data and corrected it using the local weather data. Kindly mention the RMSE if you have done. Directly depending upon Power data for assessing daily weather conditions, may not be correct especially when you are using it for further applications like optimizing crop calendars.

**Response 3.** We corrected the data using the double mass method as proposed by Bagula et al (2022). The value in terms of RMSE was acceptable since very low. As mentioned in the paper, the region is characterized by lack of data due to lack of weather station. In such a case region, downloading and correcting online data is advised. Further details on weather data handling have been added to the manuscript.

**Comment 4.** There is no presentation of the traditional calendar that is in current use; this is a major omission and should be included. More details could be provided in the introduction (lines 108-116) and even a hypothetical/estimated graphic of the traditional calendar would be useful. An analysis to show how the new calendar improves on the old one would also be useful.

**Response 4.** The manuscript has been revised accordingly to refer to current/traditional yam crop calendar though this one is not consensual among farmers. Details have been added to the introduction. A hypothetical/estimated graphic of the traditional calendar has been provided and how the new calendar improves on the old one.

**Comment 5.** The discussion is somewhat repetitive but does not include what will happen in the future e.g. 2040s, 2050s, 2080s and end of the century, when rainfall will likely be very different from the changes noted between 1990 and 2022.

**Response 5.** Thank you for this comment. As already highlighted in the document, the current manuscript relied on past data to propose a crop calendar and recommended further modeling using future scenarios to improve long term reliability of the proposed crop calendar. The manuscript has been slightly revised to emphasize such recommendation for future studies.

**Comment 6.** There is no key explaining the plot of the charts. For example, in figures 2-4, the authors could explain what each graph represents. Please provide a key for each panel of graphs. A key similar to that given in figure 5 would be useful.

**Response 6.**Thank you for this comment. This concern has been addressed accordingly. We added the explanation of some missed abbreviations and the S1 Table for variables’ description. The interannual variation is given by the line graph with the small circles, the data series’ trend by the dotted line and the fitted linear trend by the solid line to detect whether a particular index is following an increasing or a decreasing trend over time.

**Response to Reviewer #1**:

**Comment 1.**Is the Manuscript technically sound and of the data support the conclusions?

• Overall authors try to present sound basis for paper, but some gaps exist. The focus of the study is too narrow to make it useful and applicable beyond the study area.

o Efforts should be made to make comparisons with other areas in the DR, in Africa and other developing states

**Response 1.** Thank you for this useful comment and for supporting the relevance of our research. This study was conducted in South-Kivu, a very diverse region in terms of agro-ecological conditions. Though the study seems narrow-focused, calendar development procedures and suggested adaptation measures are relevant to several other parts of the country and the world with related climatic realities. Scarcity of data reported for DRC is a reality in other parts of the world when yam, a neglected and underutilized crop, is concerned. To our knowledge, this is the first report on yam crop calendar in the country and in the region in general. We hope that this study will inspire scientists and decision-makers across the continent and the world to develop yam crop calendars suiting climate change in their respective agro-ecologies. The manuscript has been revised to highlight potential broad relevance of this study’s conclusions. Whenever possible, comparisons were done with other areas, Africa, and other developing states.

**Comment 2.** Some focus/ comparison should be made with other root crops and perhaps provide a contrast with an above ground crop.

**Response 2.** As noted in the introduction, this study complements previous efforts that simulated crop calendars for staple crops in DRC. These included maize, cassava, and soybean. Whenever possible, we referred to these other crops in the manuscript.

**Comment 3.** Model Justification: Clearer justification of why CROPWAT was used over another model. For example, AquaCrop have been used to do the very same analysis.

**Response 3.** Thank you dear reviewer for this comment. Several reasons motivated the choice towards CROPWAT: (1) convenience, (2) outcome comparisons since crop calendars for other staple crops in the region were developed using CROPWAT, (3) CROPWAT is the most widely used for determining crop calendar while AquaCrop is mainly used to assess the productivity of crops such as rice, maize, sorghum, etc. This information has been added to the revised manuscript.

**Comment 4.** Traditional calendar: There is no presentation of the traditional calendar that is in current use; this is a major omission and should be included. More details could be provided in the introduction (lines 108-116) and even a hypothetical/estimated graphic of the traditional calendar would be useful. An analysis to show how the new calendar improves on the old one would also be useful.

o A comparison of the calendars could be presented as a figure 6b, or figure 7.

o Provide more details on calendar use (for any other crop) in Africa or the DRC.

**Response 4.** The manuscript has been revised accordingly to refer to current/traditional yam crop calendar though this one is not consensual among farmers. Details on calendar use on yam have been added to the introduction. A hypothetical/estimated graphic of the traditional calendar has been provided and how the new calendar improves on the old one.

**Comment 5.** The conclusion does not follow logically from the analysis. The authors claim that they sought to reduce yam vulnerability to climate change by developing a crop calendar, but this intervention only addresses rainfall. There is no discussion on what will happen in the future e.g. 2040s, 2050s, 2080s and end of century, when rainfall will likely be very different from the changes noted between 1990 and 2022. More due to other environmental changes under climate change, future water demand will change.

o For example studies have shown that because atmospheric CO2 will increase, some crops may not need to open their stomata as long (per unit time) so water loss (and hence crop water demand) could be less.

**Response 5.** Thank you for this insightful comment. Water is the most limiting factor in tropical agriculture such that the crop calendar is established based on rainfall distribution. The model used incorporated other soil, climate and crop factors while calculating water requirements (Table 1, Equations 1 & 2). CROPWAT does not, however, consider future climate data. We recommended future studies looking at the effects of future climate scenario to refine the proposed calendar. At present, there is need to specify climate models that accurately represent the province's climatic conditions, and then use data for the prediction.

**Comment 6**. The authors noted the exclusion of future period analysis (lines 623-630), but there are models and studies that have been done and can be consulted via the latest IPCC reports for the African region.

**Response 6.** Thank you for this guidance. We have read the IPCC report and publications on the African region. Unfortunately, they deal with crops (mainly cereals) and not with neglected or underutilized crops such as yam. That's why we recommended future studies involving the effect of climate change on crop calendar optimization strategies. Our team is still calibrating models to decide which one are appropriate for yam and our region.

**Comment 7.** Other key areas that could be examined are:

o Impact on crop yield as water demand changes; Impact of pest as future water availability (including drought) could become more challenging;

**Response 7.** Thank you for the comment. We have added these key areas as recommendations for future studies.

**Comment 8.** Further work is needed on the analysis: The authors have made an attempt to present data to support their claim, but more work is needed for it to make a compelling case.

o Climate trends: It would be useful to do an assessment of changes in climate over a longer period perhaps 50 years (1970-2020) and also to compare the changes in prior decades with the one in question. For example an earlier baseline period could be used (say 1961-1990) and changes before and after this be compared to see the magnitude of the changes and whether there is a continued linear trend.

**Response 8.** Thanks for the feedback. It would of course be useful to do such an analysis, but with the limited data at our disposal, we have only used the time series available to us. The only daily data available for eastern DRC are from 1970-2020. Since the crop calendar is best simulated with daily data, we decided to work with existing data series. The strength of this study lies in the fact that we used regional averages rather than a single year data to ensure that the calendar produced reflects the normal trend in climatic data for the region.

**Comment 9.** Using additional graphs to depict changes of other decades would be useful.

**Response 9.** Thank you dear reviewer for this suggestion. These results were put in the supplementary materials (Fig S4 & S5).

**Comment 10.** It would also be useful to compare the changes noted in the study region with climate of other African countries and other areas in the region. Is it a typical or expected change? Does the region represent a microclimate? Limitations: as the data used is not actual station data (but grided data corrected by station data), the authors should explain the limitations of this approach, including the effect of distance of the station from the study area, the number of stations used, challenges with bias correction.

**Response 10.** Data were analyzed to guide in crop calendar elaboration, meaning that we needed to focus on local data to ensure the calendar fits existing weather conditions. Changes are actually those experienced the last three decades and not those expected for the region. Since the region is diverse, conclusions from this study could be extrapolated to other areas with similar climates. In areas where field data are scarce, it is encouraged to use online data and correct them with existing weather stations. More details have been added on the source of data and how they were corrected.

**Comment 11.** Temperature and rainfall are distinctly different variables. One is stochastic (rainfall) while the other is continuous (temperature). For this reason, it is more useful to represent rainfall as relative (percentage change), but temperature absolute change (e.g. 0.5°C increase or decrease). For all intent and purposes a 12%increase in rainfall is not comparable to the same change in temperature since the quantities and baselines are demonstrably different.

**Response 11.** Thank you for this insightful comment, we have considered these particularities for temperature and rainfall in the revised manuscript. However, it is not practical to dissociate temperature and rainfall while assessing climate change since they are interrelated. It is why most of the time they have been combined in same formulas to elucidate effect of climate change. See Fig S4 – S10.

**Comment 12.** Charts: There is no key explaining the plot of the charts. For example, in figures 2-4, the authors could explain what each graph represent. The user is left wondering what each plot in the series represent. One intuitive view would be to interpret that the interannual variation is given by the line graph with the small circles, the mean by the dotted line and the trend by the solid line. Or is it something else? Please provide a key for each panel of graphs. A key similar to that given in figure 5 would be useful.

**Response 12.** Thank you for this observation, we have applied the necessary corrections to clarify these charts’ contents. The interannual variation is given by the line graph with the small circles, the data series’ trend by the dotted line and the fitted linear trend by the solid line to detect whether the trend is increasing or decreasing over time for a particular variable.

**Comment 13.** Suggest using a different colour gradient (maybe different colours) for table 2. This would make the difference (high, medium and low) favourable planting dates more readily discernible.

**Response 13.** Thank you for the comment. This concern has been addressed accordingly.

**Specific comments:**

**Comment 14.** Lines 46 and 60: Decide whether the term “rainfed” will be hyphenated or not and be consistent.

**Response 14.** Thank you dear reviewer this was corrected accordingly.

**Comment 15.** Line 155: Fig 1. Explain the descriptions in brackets, ie, low, medium etc.

**Response 15.** Corrected accordingly

**Comment 16.** Line 205 onward: What was the yield response factor? What was the critical depletion fraction (depletion level) used?

**Response 16.** Thank you for this comment. Limited data exist for yam. We therefore adapted the encoded data for sweet potato by coupling it with some locally available data. Indeed, the yield response factor was maintained at 0.25 and the critical depletion fraction at 0.45 (See S1 Fig.). This information has been added to the revised manuscript.

**Comment 17.** Line 211: is “P” monthly precipitation? If so, state that.

**Response 17.** Corrected accordingly

**Comment 18.** Lines 229-233: The relevance in that section is not seen. Could be placed in the Introduction or section on Method under Climate.

**Response 18.** Dear reviewer, thank you for that observation. However, this section was purposively presented there to justify the rationale behind test planting date scenarios that were selected not only based on weather data but also based on indigenous knowledge and microclimates.

**Comment 19.** Line 255 Table 1- there is a typo. Depletion is currently “deplection”.

**Response 19.** Corrected accordingly

**Comment 20.** Add acronym TAW to table 1.

**Response 20.** Corrected accordingly

**Comment 21.** Line 330-335- What is the range of the annual rainfall?

**Response 21.** Addressed accordingly

**Comment 22.** Lines 337-345- Present the range of annual rainfall with minimum and maximum temperatures.

**Response 22.** Addressed accordingly

**Comment 23.** Line 442: Check Table number.

**Response 23.** Crosschecked. It is referring to Table S2 not Table 2

**Comment 24.** Line 432: Not seeing AEZ 4 in Table 2. Was a reason presented?

**Response 24.** Corrected accordingly (See the 5^th^ row)

**Comment 25.** Line 519: Check grammar.

**Response 25.** The sentence has been sectioned for clarity.

**Comment 26.** Would have liked to see a discussion of the relationships between the soil properties and the moisture levels especially in AEZ 4.

**Response 26.** Thank you for this remark. A discussion of the relationships between the soil properties and the moisture levels especially in AEZ 4 have been added to the revised manuscript.

**Response to Reviewer #2:**

**General comment.** The authors have conducted a very relevant research, optimizing the crop calendar for Yam crop, appreciating the re effort, however, there are some minor errors which needs to be corrected.

**General response.** Dear Reviewer, We are thankful for the time you spent reviewing our manuscript. Thank you as well for supporting its relevance for publication. All the concerns you have raised have been addressed in the revised manuscript.

**Comment 1.** In the Abstract : Use bias correction was carried out, instead of Edited and Corrected using local weather stations’ records.

**Response 1.** Corrected accordingly

**Comment 2.** droughts coinciding with yam critical growth phases, instead use coinciding with critical growth phases of yam , there are such grammatical errors which needs attention through out the manuscript. Kindly rectify them.

**Response 2.** Thank you for the observation. The grammatical error has been corrected accordingly

**Comment 3.** You have mentioned that you have used NASA power data and corrected it using the local weather data. Kindly mention the RMSE if you have done. Directly depending upon Power data for assessing daily weather conditions, may not be correct especially when you are using it for further applications like optimizing crop calendars

**Response 3.** We corrected the data using the double mass method as proposed by Bagula et al (2022). The value in terms of RMSE was acceptable since very low. As mentioned in the paper, the region is characterized by lack of data due to lack of weather station. In such a case region, downloading and correcting online data is advised. Further details on weather data handling have been added to the manuscript.

**Comment 4.** In fig. 5, mention unit of measurement in the Y axis value

**Response 4.** Corrected accordingly

**Comment 5.** Fig. 6. . In the Simplified yam crop calendar Kindly mention in detail, the term ‘ crop management’ in detail, it includes Planting and harvesting too, more over there are other color codes in the fig, which are not mentioned in the legend, Kindly add them in the legend

**Response 5.** Corrected accordingly. We added the sentence that stipulate that darker areas are those that are most suitable the proposed farm activities for a same color.

**Comment 6.** In the discussion section….the word ‘report’ can be replaced by article … It is noteworthy that this report has not included

**Response 6.** Corrected accordingly

**Comment 7.** Discussion is lengthy, with repetitive contents of results, that can be removed. Such as ….

An in-depth assessment of the climate risks associated with each planting date scenario was 573 carried out. This included an analysis of rainfall, temperatures, and other climate factors bearing influence on successful yam cultivation……

**Response 7.** Thank you dear reviewer for this feedback. We shortened the section as per your comment

**Comment 8.** Appreciating the authors to add one or more pictures of the yam crop in the field settings in the annexures/ supplementary files

**Response 8.** Thank you for this suggestion, pictures have been added accordingly
